# Interposition of the transverse ligament of the knee into a fracture of the tibial plateau: a case report

**DOI:** 10.1007/s00256-018-2883-y

**Published:** 2018-02-01

**Authors:** Kazimierz T. Szopinski, Pawel Adamczyk

**Affiliations:** 1 Gamma Medical Center, Broniewskiego 3, 01-785 Warszawa, Poland; 2 Department of Dental and Maxillofacial Radiology, Faculty of Medicine and Dentistry, Medical University of Warsaw, Nowogrodzka 59, 02-006 Warszawa, Poland

**Keywords:** Tibia, Trauma, Transverse ligament of the knee, Interposition

## Abstract

Interposition of the transverse ligament of the knee between fragments of an intercondylar eminence fracture was diagnosed using magnetic resonance imaging (MRI) in a 11-year-old boy after a sports injury. The interposition was confirmed and corrected during arthroscopy. We report what we believe to be the first published case of isolated interposition of the transverse ligament in a minimally displaced fracture of the tibial eminence.

## Introduction

Transverse ligament (TL) of the knee, also known as anterior intermeniscal ligament (AIL) or transverse geniculate ligament is an anatomical structure inserting into the anterior horns of the menisci [1, 2]. It is located posterior to Hoffa’s fat pad and may occasionally be visible on lateral plain radiographs of the knee [3]. However, the imaging method of choice of the transverse ligament of the knee is magnetic resonance imaging (MRI). The reported incidence of the TL ranges between 31 and 94% of the population (Table 1) [1–8]. The TL restricts the antero-posterior excursion of the medial meniscus during the early phase of knee flexion [9]. Increased tension of this ligament during knee flexion and rotation may contribute to tears of the anterior horn of the medial meniscus [2].

**Table 1:** The incidence of the transverse ligament of the knee

Reference	Method	Number of specimens / imaged knees	Incidence of the transverse ligament of the knee (%)
[4]	Cadaveric	92	69
[5]	Cadaveric	34	71
[6]	Cadaveric	50	94
[7]	Lateral radiograph	50	12
[7]	MRI	50	68
[1]	MRI	229	53
[1]	MRI + arthroscopy	36	MRI 44, arthroscopy 67
[8]	MRI	100	31
[2]	MRI	49	73.5

Sports injuries of the tibial eminence are common, and usually result from forced valgus and external rotation of the tibia or hyperflexion and internal rotation of the tibia [10]. The initial diagnosis is usually based on clinical findings and a set of knee radiographs (antero-posterior and lateral projections), or a computed tomography (CT) scan. MRI examination is mandatory, if injury to soft tissues, cartilage, or ligaments is suspected. To the authors’ knowledge this is the first published case of traumatic interposition of the transverse ligament of the knee between the bony fragments in a minimally displaced fracture of the tibial eminence.

## Case report

**Fig. 1:**
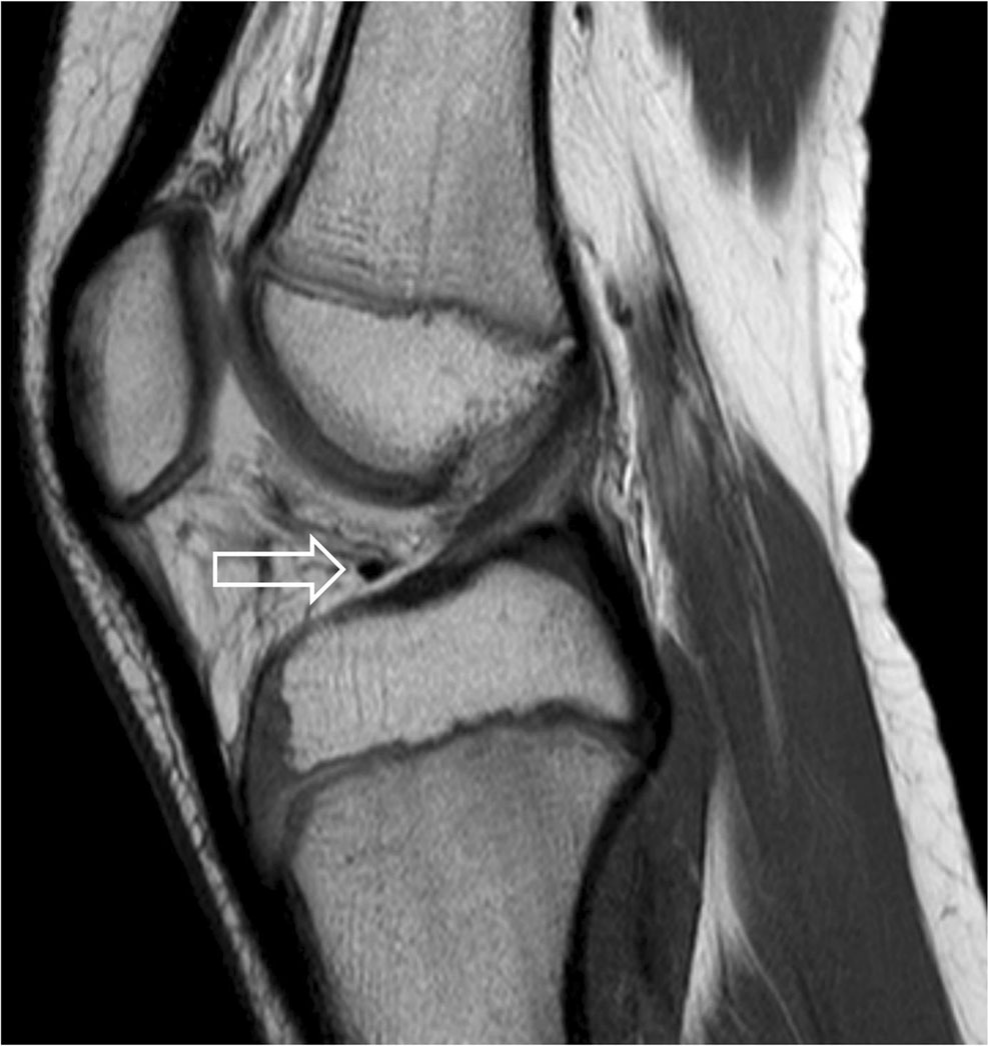
Sagittal proton density (PD)-weighted image of the knee 1 week before the accident. The normal transverse ligament is seen in its anatomical position (*arrow*). No signs of Osgood–Schlatter disease are visible

An 11-year-old boy underwent MRI at our clinic to rule out Osgood–Schlatter disease. The images were obtained with an Achieva 1.5-T MRI system (Philips Healthcare, Amsterdam, Netherlands) using a knee coil. No signs of Osgood–Schlatter disease were demonstrated, and the transverse ligament of the knee was present and normal in appearance (Fig. 1).

One week later the same patient had a sports injury—a direct impaction of the flexed knee against a boulder during a snow-board ride. Radiography performed in the emergency ward at the skiing resort demonstrated a minimally displaced fracture of the tibial eminence, and the patient’s knee was treated with a cast. The diagnosis of a minimally displaced fracture of the tibial eminence was confirmed by CT performed at another institution (not shown).

**Fig. 2:**
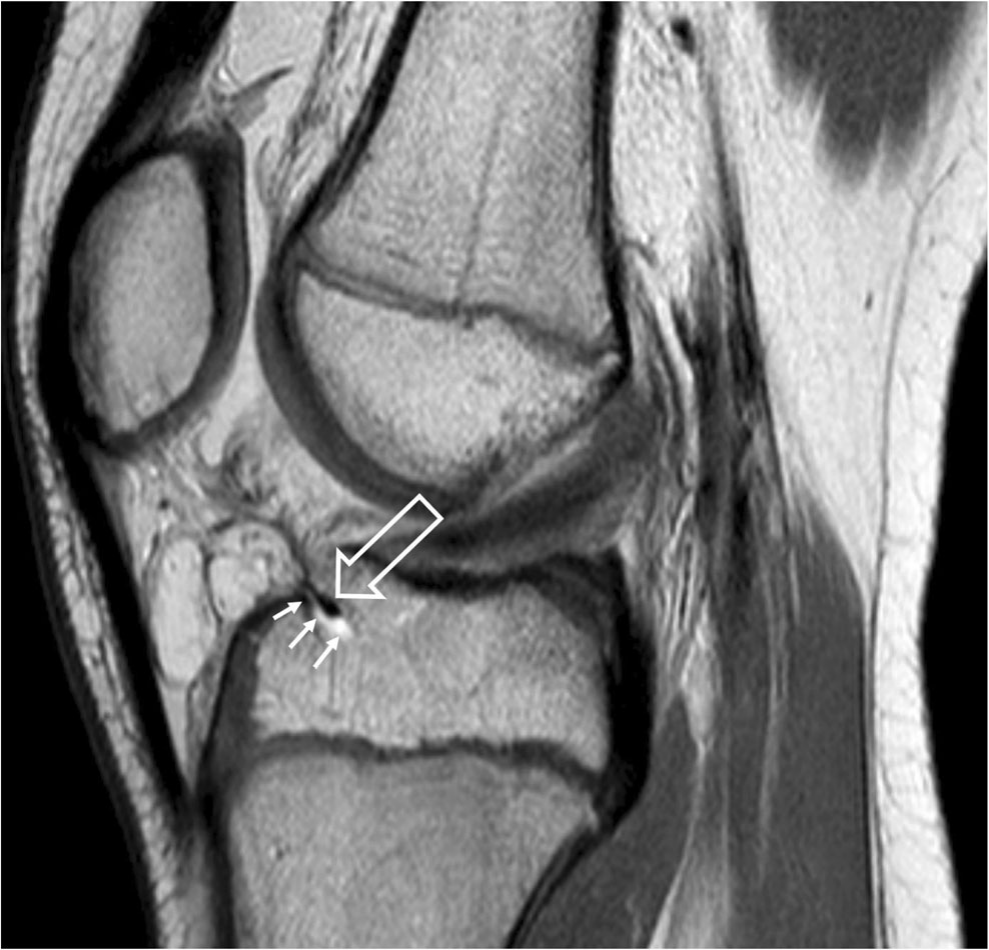
Sagittal PD image of the knee 1 week after the trauma. A fracture line in the anterior portion of the intercondylar eminence (*small arrows*) and inferior displacement and interposition of the transverse ligament of the knee between the fragments of the fractured bone is demonstrated (*large arrow*)

The patient was referred to our institution 7 days after the trauma. MRI performed on the same day, using the same machine and imaging technique as previously, demonstrated a minimally displaced fracture of the intercondylar eminence involving the articular cartilage. Additionally, inferior displacement and interposition of the transverse ligament of the knee between the fragments of the fractured bone were demonstrated (Fig. 2).

**Fig. 3:**
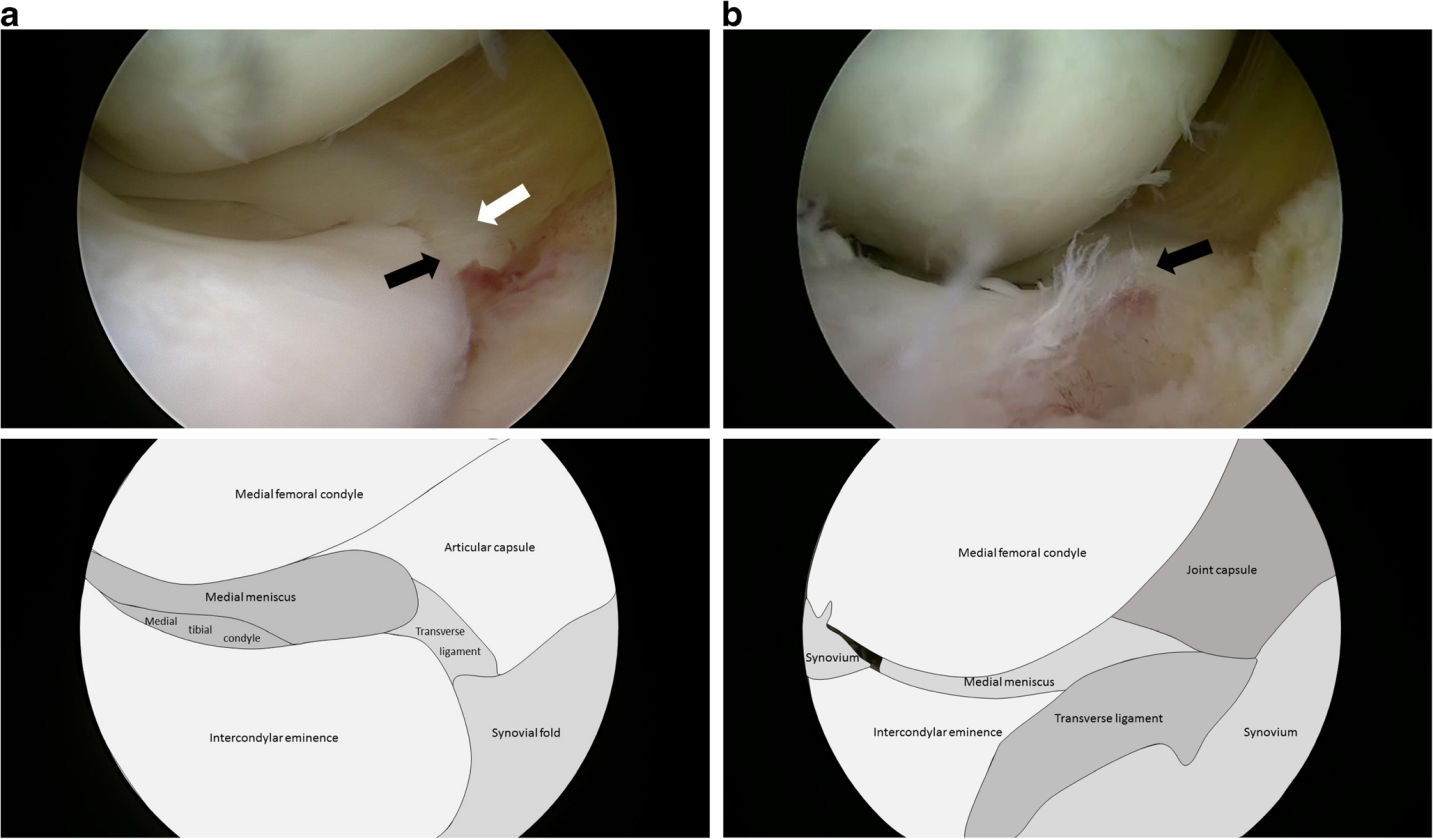
**a** Intraoperative image. Transverse ligament of the knee (*white arrow*) interposed under the tibial eminence (*black arrow*). **b** Intraoperative image. Transverse ligament of the knee (*arrow*) has been restored to its anatomical position after tibial eminence stabilization

**Fig. 4:**
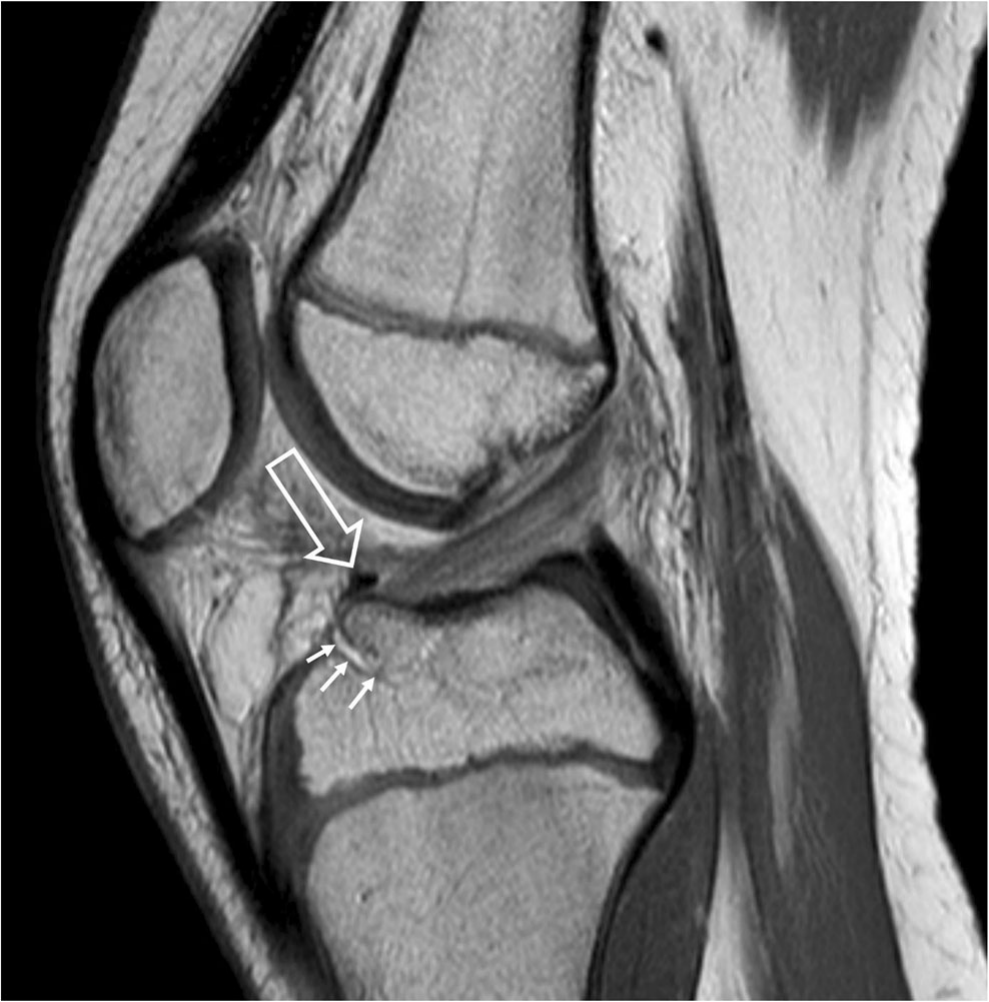
Control MR examination 6 weeks after the operation, sagittal PD image of the knee. The repositioned transverse ligament (*large arrow*) is seen in its normal position. The fracture line in the anterior portion of the tibial eminence is still visible (*small arrows*)

Eleven days after injury the patient underwent arthroscopic surgery. The intraoperative findings confirmed the radiological diagnosis (Fig. 3a). The transverse ligament was successfully repositioned and bony fragment with anterior cruciate ligament insertion was stabilized using an absorbable suture loop (Fig. 3b). A normal position was confirmed in a follow-up MRI performed 6 weeks after the operation (Fig. 4).

Over a 6-month follow-up, the patient has been pain-free and has had a full range of knee motion.

## Discussion

Fractures of the tibial eminence are common in children between 8 and 12 years of age, usually resulting from sports trauma. In most cases, these fractures present as isolated injuries.

Fractures of the tibial eminence are graded according to the modified Meyers and McKeever classification (Table 2). The original classification (three types based on the severity of displacement of the avulsed bone fragment) was proposed by Meyers and McKeever in 1959 [11]. The classification was subsequently modified by the same authors in 1970, creating a new subgroup (III+) encompassing cases with rotated bone fragment [12]. In 1977, Zaricznyj added type IV, including comminuted fractures [13]. The type I and reducible type II lesions can be treated conservatively, the unreducible type II lesions and the type III and IV lesions require surgery [9, 14]. The presented case can be classified as a reducible type II lesion.

**Table 2:** Classification of fractures of the intercondylar eminence of the tibia

	Type according to Meyers and McKeever [11]	Type according to Meyers and McKeever [12]	Type according to Zaricznyj [13]
Minimum displacement of the avulsed fragment and excellent bone apposition	I	I	I
Elevation of the anterior third to half of the avulsed fragment producing a beaklike appearance on the lateral roentgenogram	II	II	II
Avulsed fragment completely separated from its bone bed, no apposition of the fragment	III	III	IIIA
Avulsed fragment completely separated from its bone bed, and rotated so that the cartilaginous surface of the cartilaginous surface of the fragment faces the raw bone of the bone bed making union impossible	–	III+	IIIB
Comminuted fracture	–	–	IV (IIIC)

The diagnosis is usually based on history, clinical findings, and radiography. If the radiographic image is equivocal, the fracture lines and structure of the bone may be assessed in CT. However, radiography or CT, although performant in assessment of the osseous structures, has only a limited value in the diagnosis of soft-tissue injuries. The reported lesion would have been missed if the imaging were limited only to radiography or CT. In our institution MRI is a gold standard in the assessment of sports injuries of the knee, demonstrating abnormalities of both bone and soft tissues. Ultrasound would not be helpful in this particular case as the transverse ligament was positioned deeply in the fracture. Moreover, the transverse ligament of the knee is not usually assessed in routine ultrasound examinations of the knee and is absent in a large proportion of normal knees.

To the authors’ knowledge no cases of interposition of the TL into a fracture were published. Cadaveric studies show that the TL inhibits posterior translation of the anterior horn of the medial meniscus in the early degrees of the knee flexion (30°) and has no effect on the meniscal motion at extension, 60° flexion, and full flexion [9]. The disruption of the transverse ligament of the knee results in retraction of the anterior horn of the medial meniscus medially and distally over the proximal anterior tibia [6]. The increased frequency of medial meniscal tears was found in patients with a TL attachment to the medial meniscus, compared with patients without this attachment [2]. Therefore, we speculate that limited mobility of the medial meniscus in an untreated case of interposition of the TL into a fracture may result in reduced movement range of the knee and tear of the medial meniscus.

## Conclusion

Magnetic resonance imaging should be highly recommended to rule out unexpected pathological findings in patients with fractures of the tibial plateau, even in cases with minimal dislocation of fragments.
